# Draft Genome Sequence of *Criibacterium bergeronii* gen. nov., sp. nov., Strain CCRI-22567^T^, Isolated from a Vaginal Sample from a Woman with Bacterial Vaginosis

**DOI:** 10.1128/genomeA.00959-16

**Published:** 2016-09-01

**Authors:** Andrée F. Maheux, Ève Bérubé, Dominique K. Boudreau, Frédéric Raymond, Jacques Corbeil, Paul H. Roy, Maurice Boissinot, Rabeea F. Omar

**Affiliations:** a Centre de Recherche en Infectiologie de l’Université Laval, Axe Maladies Infectieuses et Immunitaires, Centre de Recherche du CHU de Québec-Université Laval, Québec City, Québec, Canada; b Département de Microbiologie, Infectiologie et d'Immunologie, Faculté de Médecine, Université Laval, Québec City, Québec, Canada; c Centre de Recherche en Données Massives, Université Laval, Québec City, Québec, Canada; d Département de Médecine Moléculaire, Université Laval, Québec City, Québec, Canada

## Abstract

*Criibacterium bergeronii* gen. nov., sp. nov., CCRI-22567 is the type strain of the new genus *Criibacterium*. The strain was isolated from a woman with bacterial vaginosis. The genome assembly comprised 2,384,460 bp, with 34.4% G+C content. This is the first genome announcement of a strain belonging to the genus *Criibacterium*.

## GENOME ANNOUNCEMENT

*Criibacterium bergeronii* gen. nov., sp. nov., CCRI-22567^T^ was isolated from a vaginal clinical swab harvested from a woman presenting a bacterial vaginosis (BV; Nugent score of 9) during a clinical study conducted in Spring 2014 at Centre Hospitalier Universitaire de Québec (Québec City, Canada). The isolated colony was subjected to PCR and DNA sequencing of the 16S rRNA gene for species identification. Comparative 16S rRNA gene sequence analysis showed that the closest cultured relative of strain CCRI-22567^T^ was *Eubacterium yurii* subsp. *schtika* ATCC 43716^T^ (88.0%) (1). The two closest organisms with an available draft genome were compared to strain CCRI-22567^T^ for genomic relatedness assessment. The genomic average nucleotide identity by BLAST (ANIb) (2) obtained with *Eubacterium yurii* subsp. *margaretiae* ATCC 43715 (accession no. NZ_AEES01000000) is 68.5% and 67.4% with *Filifactor alocis* ATCC 35896 (accession no. NC_016630). This indicates that strain CCRI-22567^T^ represents a novel species of a new genus in the *Peptostreptococcaceae* family (3, 4). The role of this new bacterium in BV remains unclear, since *Gardnerella vaginalis*, *Bacteroides fragilis*, and *Mobiluncus curtisii* (all species consistent with BV) were also isolated from the same vaginal swab.

*Criibacterium bergeronii* is a rod-shaped strictly anaerobic bacterium and the only cultured member of the genus *Criibacterium*. It grows on blood agar in 48 to 72 h at 35°C incubated under an anaerobic atmosphere, as previously described (5), forming pinpoint <1-mm translucent flat colonies. Genomic DNA was isolated by using BioSprint 15 DNA blood kit (Qiagen) automated with a KingFisher mL instrument (Thermo Fisher Scientific). Whole-genome sequencing of strain CCRI-22567^T^ was performed on an Illumina HiSeq 2500 using SBS version 4 to sequence a 126-bp paired-end library (Nextera XT; Illumina). A total of 7,787,976 reads were assembled *de novo* in 19 contigs using Ray software (version 2.3.0) (6). The total genome length is 2,384,460 bp (*N*_50_, 321,664 bp), with an average G+C content of 34.4%. The draft genome sequence was annotated using NCBI GenBank annotation pipeline (version 3.3) and RAST annotation server (version 2.0). A total of 2,324 features were identified, including 9 rRNAs and 52 tRNAs. Of the 2,173 putative coding sequences, 914 were assigned as hypothetical proteins. A comparison of the 16S rRNA gene sequence with microbiome projects available in GenBank NR database also suggests that *Criibacterium bergeronii* is present on sebaceous parts of the skin (7). The complete genome sequence will help understand its ecological association with human microbiota.

*Criibacterium bergeronii.* (Cri.i.bac.te′ri.um. N. L. n. *Cri* from CRI, the abbreviation of Centre de Recherche en Infectiologie; N. L. neut. n. *bacterium* from Gr. n. *bakterion* a small rod; N. L. neut. n. *Criibacterium* a small rod isolated at CRI). *bergeronii* (ber.ge.ron′ii N. L. masc. gen. *bergeronii* of Bergeron, named after Professor Michel G. Bergeron (CM, OQ, MD, FRCPC, FCAHS, FIDSA), infectious diseases specialist, serial entrepreneur, Founder and former Director of Centre de Recherche en Infectiologie de l’Université Laval in Québec City [Québec], Canada [8]).

### Accession number(s).

The whole-genome shotgun project of *Criibacterium bergeronii* CCRI-22567^T^ has been deposited at DDBJ/EMBL/GenBank under the accession no. MBEW00000000. The version described in this paper is version MBEW01000000.
